# Collision Mortality Has No Discernible Effect on Population Trends of North American Birds

**DOI:** 10.1371/journal.pone.0024708

**Published:** 2011-09-09

**Authors:** Todd W. Arnold, Robert M. Zink

**Affiliations:** 1 Department of Fisheries, Wildlife, and Conservation Biology, University of Minnesota, St. Paul, Minnesota, United States of America; 2 Bell Museum and Department of Ecology, Evolution and Behavior, University of Minnesota, St. Paul, Minnesota, United States of America; University of Western Ontario, Canada

## Abstract

Avian biodiversity is threatened by numerous anthropogenic factors and migratory species are especially at risk. Migrating birds frequently collide with manmade structures and such losses are believed to represent the majority of anthropogenic mortality for North American birds. However, estimates of total collision mortality range across several orders of magnitude and effects on population dynamics remain unknown. Herein, we develop a novel method to assess relative vulnerability to anthropogenic threats, which we demonstrate using 243,103 collision records from 188 species of eastern North American landbirds. After correcting mortality estimates for variation attributable to population size and geographic overlap with potential collision structures, we found that per capita vulnerability to collision with buildings and towers varied over more than four orders of magnitude among species. Species that migrate long distances or at night were much more likely to be killed by collisions than year-round residents or diurnal migrants. However, there was no correlation between relative collision mortality and long-term population trends for these same species. Thus, although millions of North American birds are killed annually by collisions with manmade structures, this source of mortality has no discernible effect on populations.

## Introduction

Habitat destruction, overexploitation, climate change and the creation of manmade obstacles have been identified as the major threats to migrating animals [1], especially birds [2]-[4]. Habitat destruction is unquestionably the most important anthropogenic threat to biodiversity [5], but habitat loss does not directly cause mortality among mobile organisms, rather displaced organisms suffer higher mortality or lower fecundity when they are forced into suboptimal habitats [6]. For North American birds, the major anthropogenic sources of mortality include predation by house cats, poisoning, and collisions with windows, communication towers, high-tension wires and motor vehicles [7]. Buildings and towers do not represent absolute barriers to avian migration in the same way that dams impede upstream movements by anadromous fish or fences restrict movements of wild ungulates [1], but such structures increase the mortality risk to migrating birds [8]. Evans Ogden [8] estimated that 70% of migrant birds in eastern North America migrate through at least one major metropolitan area during each migration event. Nocturnal migrants frequently collide with skyscrapers after becoming disoriented by building lights, especially during inclement weather [8], and birds making migratory stopovers in urban areas are especially prone to collision with low-level windows as they forage in unfamiliar environments [8]–[10]. Eastern North America also has >60,000 communication towers >60m tall and documented kills of migratory birds at individual towers have ranged from 80 to 3,200 birds per year [7], [11].

Despite widespread public attention and more than five decades of research, measures of anthropogenic sources of mortality for North American birds remain speculative. Estimates of total collision mortality from communication towers in North America range from 0.94 to 50 million birds annually [11], [12], whereas estimates of collision mortality with windows range from 3.5 million up to 5 billion birds annually [9], [10], [12]. Using the most frequently cited median estimates of 25 million mortalities from collisions with towers [11] and 1 billion mortalities from collisions with windows [9], these two mortality sources in aggregate represent 21% of the estimated breeding population of 4.9 billion North American landbirds [13].

In this paper, we develop a novel method for assessing the potential impact of a given mortality source on population dynamics that does not require a precise estimate of total mortality, which would be difficult if not impossible to obtain for most migratory wildlife. Our method uses comparative data, including species-specific measures of mortality, relative abundance, and long-term population trends. We utilize long-term records of avian mortality from communication towers [14] and urban buildings [8] combined with population estimates and trend data from the North American Breeding Bird Survey [13], [15] to test the hypothesis that these two mortality sources have influenced population trends among North American birds.

## Results

**Figure 1:**
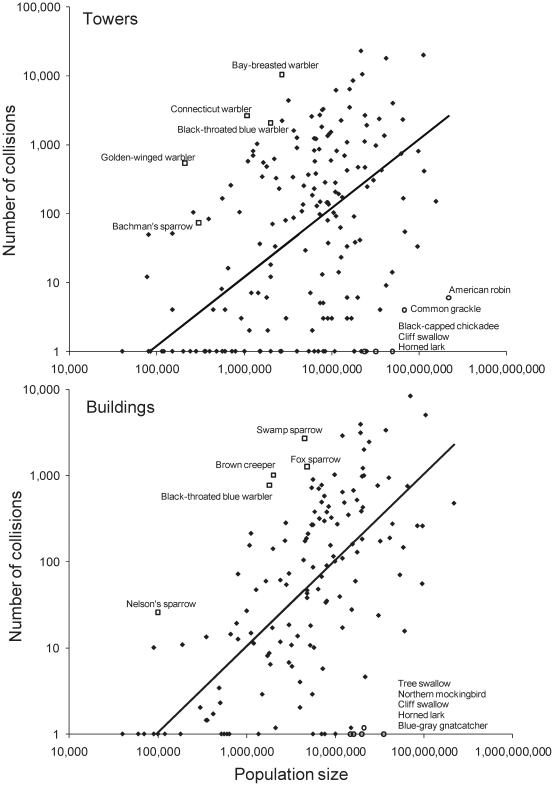
Collision mortality of eastern North American landbirds with towers and buildings as a function of population size. Data are from 39 communication towers (top) and three sets of urban buildings (bottom) and each datum represents a single species. Trend lines pass through geometric mean (X,Y) with a slope of 1, so that residuals are measures of relative mortality risk to tower and building collision. The top 5 “super colliders” and “super avoiders” on each figure are labeled; for avoiders the lists from top to bottom correspond to open symbols from left to right along the X-axis.

Our analysis was based on 180,832 mortalities of 188 species from 39 different communication towers and 62,271 mortalities of 147 species from three different cities for building collisions (Figure S1). For both communication towers and buildings, there were five species that comprised >5% of the total mortalities, with only one species (ovenbird, *Seiurus aurocapillus*) among the top five in both data sets. However, total body counts reveal little about relative mortality risk for each species because they are potentially confounded by variation in population size (range: 40,000 to 220 million) and for towers by potential range overlap with monitored collision sites (range: 2 to 39). The correlation between log_10_ building mortalities (m) and log_10_ population size (N) was highly significant (*r* = 0.57, *P*<0.0001, *n* = 147), as was the correlation between log_10_ tower mortalities and log_10_ population size (*r* = 0.43, *P*<0.0001, *n* = 188) (Figure 1). Tower collisions were additionally affected by overlap with collision sites (S); multiple regression: log_10_(m+1)  =  -3.52[SE 0.73] + 0.54[SE 0.35]*log_10_(N) + 1.06[SE 0.35]*log_10_(S) (*R* ^2^ = 0.22, *F*_3,186_ = 26.12, *P*<0.0001). To estimate relative vulnerabilities among species, we forced regression coefficients for population size and site overlap to their theoretical values of +1.0 [16] and calculated residuals for each species: relative vulnerability  =  log_10_(m+1) – Y – [log_10_(N+1) – X_1_] - [log_10_(S+1) – X_2_], where Y is mean log_10_ mortalities (buildings: 1.672, towers: 1.698), X_1_ is mean log_10_ population size (buildings: 6.651, towers: 6.618), and X_2_ is mean log_10_ collision sites (1.539; towers only).

These residuals represent relative collision vulnerability, which varied enormously among species. For buildings, the top five species (“super colliders”) were 25 to 57 times more likely to collide with a building than expected by chance (Fig. 1; Table S1). By contrast, the bottom five species of super avoiders were 105 to 208 times less likely to collide with a building than expected by chance. For towers, super colliders were 96 to 236 times more likely to collide than expected by chance, whereas super avoiders were 222 to 688 times less likely to collide. Only two species identified as super colliders based on relative collision vulnerability were also among the top five species based on simple body counts (swamp sparrow at buildings, bay-breasted warbler at towers; for scientific names see Table S1). Black-throated blue warblers were super colliders at both buildings and towers, whereas cliff swallows and horned larks were super avoiders for both structure types.

There was a strong taxonomic component to collision vulnerability. For 13 avian families that contributed five or more species to the tower collision data, relative vulnerability varied significantly among families (*F*_12,132_ = 12.47, *P*<0.0001) with wood warblers (Parulidae, *n* = 36) averaging 18-fold greater vulnerability and swallows (Hirundinidae, *n* = 6) averaging 54-fold lower vulnerability (Table S2). Taxonomic effects were also prominent for building collisions (*F*_12,101_ = 9.88, *P*<0.0001), with wood warblers and swallows once again representing the extremes. Although there were notable differences between towers and buildings in relative vulnerabilities for some species, residuals from the two data sets were highly correlated (*r* = 0.60, *P*<0.0001, *n* = 147; see Figure S2) indicating that most species had similar vulnerability to both mortality sources.

Vulnerability to tower collision varied according to migration distance (*F*_2,185_ = 16.64, *P*<0.0001). Species migrating long distances to the Neotropics were 17.3 times more likely to collide with towers than were non-migrants, whereas short-distance migrants were 2.3 times more likely to collide. Migration strategy played a lesser role for building collisions (*F*_2,144_ = 3.53, *P* = 0.03), with Neotropical and short-distance migrants being 3.5 and 2.4-fold more likely to collide with buildings than non-migrants. Timing of migration flights was also important for both data sets (towers: *F*_2,165_ = 36.71, *P*<0.0001; buildings: *F*_2,131_ = 31.40, *P*<0.0001), with nocturnal migrants averaging 30.1 (towers) and 10.9 (buildings) times greater vulnerability than diurnal migrants

**Figure 2:**
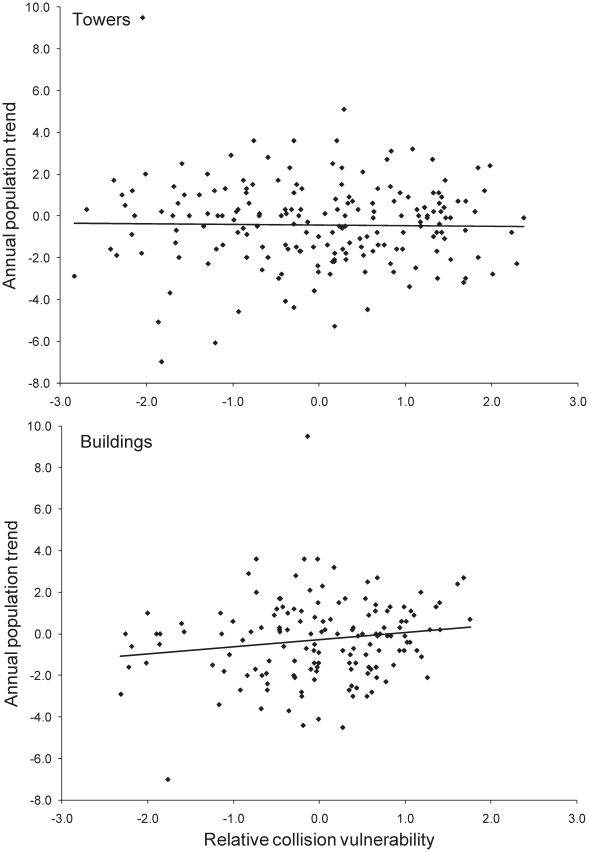
Long-term annual population trends of eastern North American landbirds versus relative collision vulnerability with towers and buildings. Data are from communication towers (top, *n* = 177) and buildings (bottom, *n* = 138), with each datum representing a single species. No trend data were available for 11 mostly far-northern species. One outlier not shown in top graph (Bewick's wren: 0.32, −12.6), but trend line and statistics include this species.

Most surprisingly, for 177 species with available data on population trends, there was no correlation between relative collision vulnerability with communication towers and annual rate of population change (Figure 2: *r* = −0.017, *P* = 0.83). For buildings there was a weak positive correlation, such that species that collided most frequently with buildings were more likely to exhibit population increases (Figure 2: *r* = 0.154, *P* = 0.06, *n* = 138). We acknowledge that collision mortality is unlikely to be the sole source of population decline, but our analysis had substantial power to detect even partial effects of collision mortality on population change. Using observed variances and sample sizes from the tower and building analyses, respectively, we had 80% power to detect real annual population declines of 0.35 or 0.47% per year associated with 10-fold increases in collision mortality. Alternatively, our analyses had 80% power to detect factors that explained as little as 4.1% of the observed variation in population trends for tower data and 5.2% of the variation for building data.

## Discussion

Although migrant birds were at greater risk of collision mortality with buildings and towers, our analyses suggest that this conspicuous source of mortality has had no discernible effect on long-term population dynamics among North American landbirds, and this finding was robust to all of the statistical adjustments that we employed. At worst, collision mortality could be described as an added burden for populations already in decline for other reasons, and our list of super colliders (Table S1) includes two such species: golden-winged warbler and Bachman's sparrow, but the list of super colliders also includes two species that are increasing significantly [15].

Simply documenting what species are killed and in what frequencies does not identify whether a particular threat constitutes an additive source of mortality. Even if only 100 million North American birds die annually from collisions with structures [7]-[9], it would seem to be a serious source of mortality. However, many birds are abundant and have high reproductive potentials. For example, ovenbirds are among the top species encountered at most collision sites [8], but the population estimate for ovenbirds is 24 million [13]. At one of the longest monitored collision sites, 15,987 ovenbirds collided with a TV tower during a 38-year monitoring period [17], for an average of 420/yr. To reach a 10% annual mortality level, ovenbirds would need to encounter over 5,500 sites like this tower, but average kill of ovenbirds at several other sites we examined was ∼80 per year. And even this level of mortality might not have population consequences; North American waterfowl hunters annually harvest 10–15% of the fall mallard (*Anas platyrhynchos*) population with only modest impact on subsequent population size [18]. Most avian collision mortality occurs during fall migration [8], [11], [19], when annual populations are at their zenith. If bird populations are ultimately limited by habitat availability [6], collision mortality will be largely compensatory to natural sources of mortality.

These conclusions need not prevent the deployment of simple design solutions that can greatly reduce avian collision mortality at manmade structures. In particular, shorter towers with flashing lights can minimize collision risk at communication towers [20], [21], whereas turning out lights during peak migratory periods and minimizing vegetation near glass building faces can minimize collisions with buildings [22], [23]. Nevertheless, our analysis suggests that such activities will not halt population declines among North American migratory birds.

The methods we developed in this analysis are well suited to birds due to continental monitoring efforts that provided detailed information on population size and trends [13], [15], but the methods could be easily modified for less-studied taxa using categorical trends (i.e. increasing, stable, declining). We believe that many poorly quantified conservation threats, ranging from topics as diverse as house cat predation [24], wind towers [11] and bushmeat consumption [25], could be assessed using similar methodology.

## Materials and Methods

Our analysis was based on existing summaries of birds that accidentally killed themselves by colliding with towers or buildings, and does not require approval by an Institutional Animal Care and Use Committee under official guidelines of the University of Minnesota.

Tower collision data were obtained from a summary of 47 studies from 39 separate sites in eastern North America [14]. We compiled data on landbird collisions with urban buildings from three unpublished data sets: Chicago, Illinois (D. Willard, pers. comm.) [26]; Toronto, Ontario (www.flap.org) [8]; and New York, New York (www.nycaudubon.org) [23]. We assume that monitored sites were representative of potential collision sites throughout eastern North America. Sites were not randomly selected, but monitored towers were widely dispersed throughout eastern North America and relative collision vulnerability was consistently and positively correlated between pairs of towers (see Figure S1, Appendix S1). For the three building sites, correlations of relative collision vulnerability among sites were 0.47, 0.59 and 0.83. Although more data on window collisions would have been desirable, we believe our data to be broadly indicative of collision mortality at any random sample of towers or buildings in eastern North America. Moreover, the strong correlation between tower vulnerability and building vulnerability suggests that we have adequately measured collision risk for most species (Figure S2).

Our methods assume no pronounced biases among species in carcass detection probabilities. Previous studies have documented underestimation of collision mortality due to failure to find carcasses in dense vegetation, scavenging of carcasses by predators and movement away from collision sites by injured birds [7], [8], [11], [23]; however, we are not aware of any studies that have shown detection biases to vary among species. We presume there may be modest detection biases in carcass surveys (see Appendix S1), but that they are trivial in comparison to the massive differences in relative collision vulnerability that we documented.

For communication towers, we quantified the number of collision sites that were within the breeding, migratory or wintering range of each bird species for use as a covariate [14], [27], [28]. For buildings, we restricted the data to species occurring regularly at all three collision sites [27], [28] and standardized each site to 20,000 total mortalities to give each site equal weighting. We used range maps to categorize migration strategy as long-distance (Neotropical), short-distance or partial/non-migratory [27], [28]. Data on timing of migration (primarily diurnal, primarily nocturnal, both or unknown) were obtained from *Birds of North America* species accounts [28].

Estimated population sizes [13] were based on the North American Breeding Bird Survey (BBS) adjusted for the proportion occurring east of 100° longitude (for tower analyses) or east of the Mississippi River (for buildings). For the tower and building data we also included 38 and 16 species, respectively, that were not recorded as mortalities but were at risk based on range overlap with collision sites [27], [28]. Population trends (1966–2009) were based on recent hierarchical models for the entire eastern BBS region [15], which was our area of inference for collision mortality. Although there is undoubtedly sub-regional variation in population trends among some species of eastern North American landbirds, 55.8% of the total variation in population trends among 11 different Bird Conservation Regions (BCRs) was attributable to species (nested ANOVA, *F*_168,1153_ = 10.89, *P*<0.0001), with the remaining 44.2% of variation comprising real regional variation plus sampling variation. Our sample of 39 communication towers was distributed throughout the entire eastern BBS region (Figure S1), but our analysis of building sites was more limited so we conducted additional analyses based only on the Bird Conservation Regions that included Chicago, Toronto, and New York and obtained similar results (Appendix S1).

To derive species-specific measures of per-capita collision risk we regressed log_10_ total collision mortality (using log_10_(X+1) to account for zeros) against log_10_ population size to derive species-specific measures of per-capita collision risk. Because there was unknown error in both Y and X, neither ordinary least squares (OLS) nor reduced major axis (RMA) regression provides an unbiased estimate of the true slope [16]. Instead we fixed the slope at its theoretical value of +1.0 (i.e., all other things being equal, a 10-fold increase in abundance should lead to a 10-fold increase in collision mortality) and used OLS and RMA regression for verification. We used a similar approach for tower data by concurrently regressing log_10_ mortalities against log_10_ population size and log_10_ number of collision sites exhibiting range overlap with the species in question. We treated species as statistically independent data points for most analyses and analyzed data using general linear models in Program SAS [29]. For analyses involving ecological covariates (e.g., migration distance) that might be similar among closely-related species due to shared evolutionary histories, we used phylogenetic methods [30] (see Appendix S1).

## Supporting Information

Figure S1 **Map of approximate locations of communication towers (red dots) and downtown buildings (larger blue dots) used in assessment of collision mortality for eastern North American landbirds.** Tower sites were redrawn from Shire et al. [14].
(PDF)

Figure S2 **Relative vulnerability to collision with towers (i.e., residuals from** **Fig. 1a** **) versus buildings (** **Fig. 1b** **).** With the exception of 3 labeled outliers that had modest vulnerability to buildings but avoided towers, collision risk at towers was a good proxy for collision risk at buildings, and vice versa.
(TIFF)

Table S1 **List of North American landbird species that collide most frequently with towers (T1-T5) and buildings (B1-B5) (the “super colliders”), as well as the top five super avoiders (T188-184, B147-143).**
(PDF)

Table S2 **Taxonomic variation in collision risk for families of North American birds.**
(PDF)

Appendix S1 **Supplementary Analyses.**
(PDF)
