# Supplementary figures and images for: Collision Mortality Has No Discernible Effect on Population Trends of North American Birds
Source: PLoS One. 2011 Sep 9;6(9):e24708. doi: 10.1371/journal.pone.0024708 (PMC3170378; doi:10.1371/journal.pone.0024708)

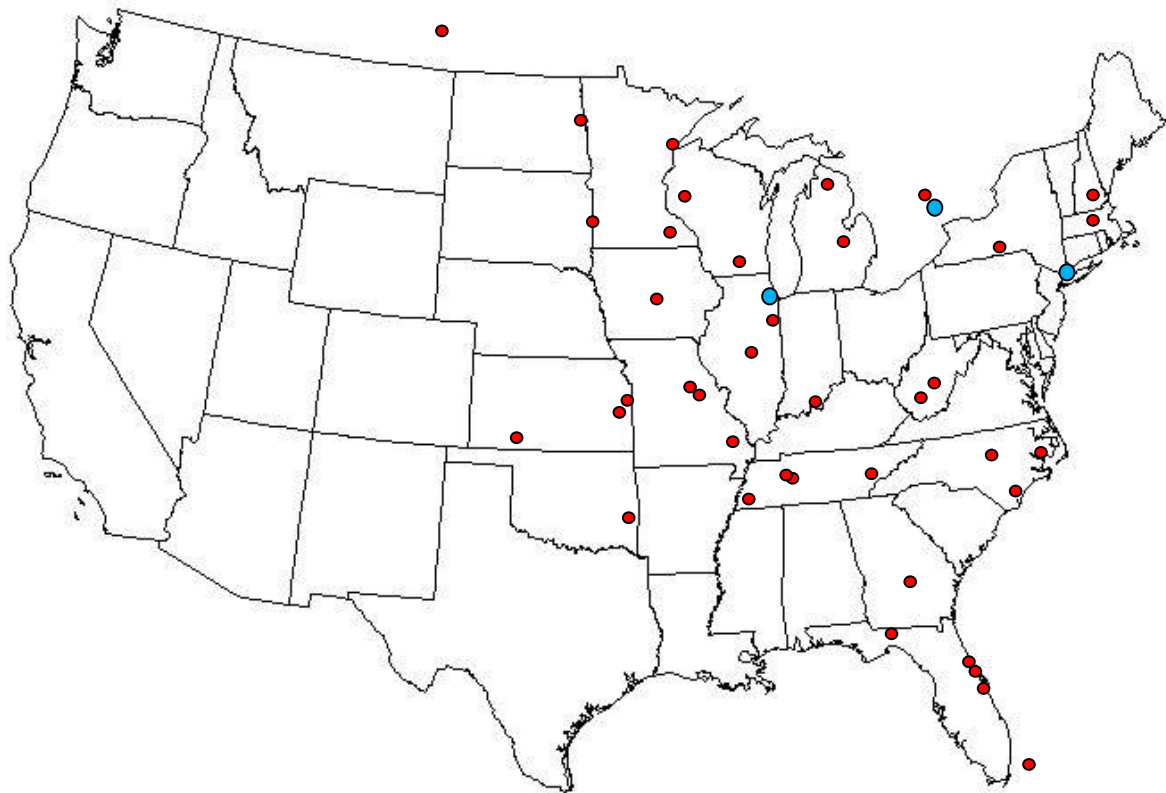

Supplement: Figure S1 — Map of approximate locations of communication towers (red dots) and downtown buildings (larger blue dots) used in assessment of collision mortality for eastern North American landbirds. Tower sites were redrawn from Shire et al. [14]. (PDF) [file pone.0024708.s001.pdf]

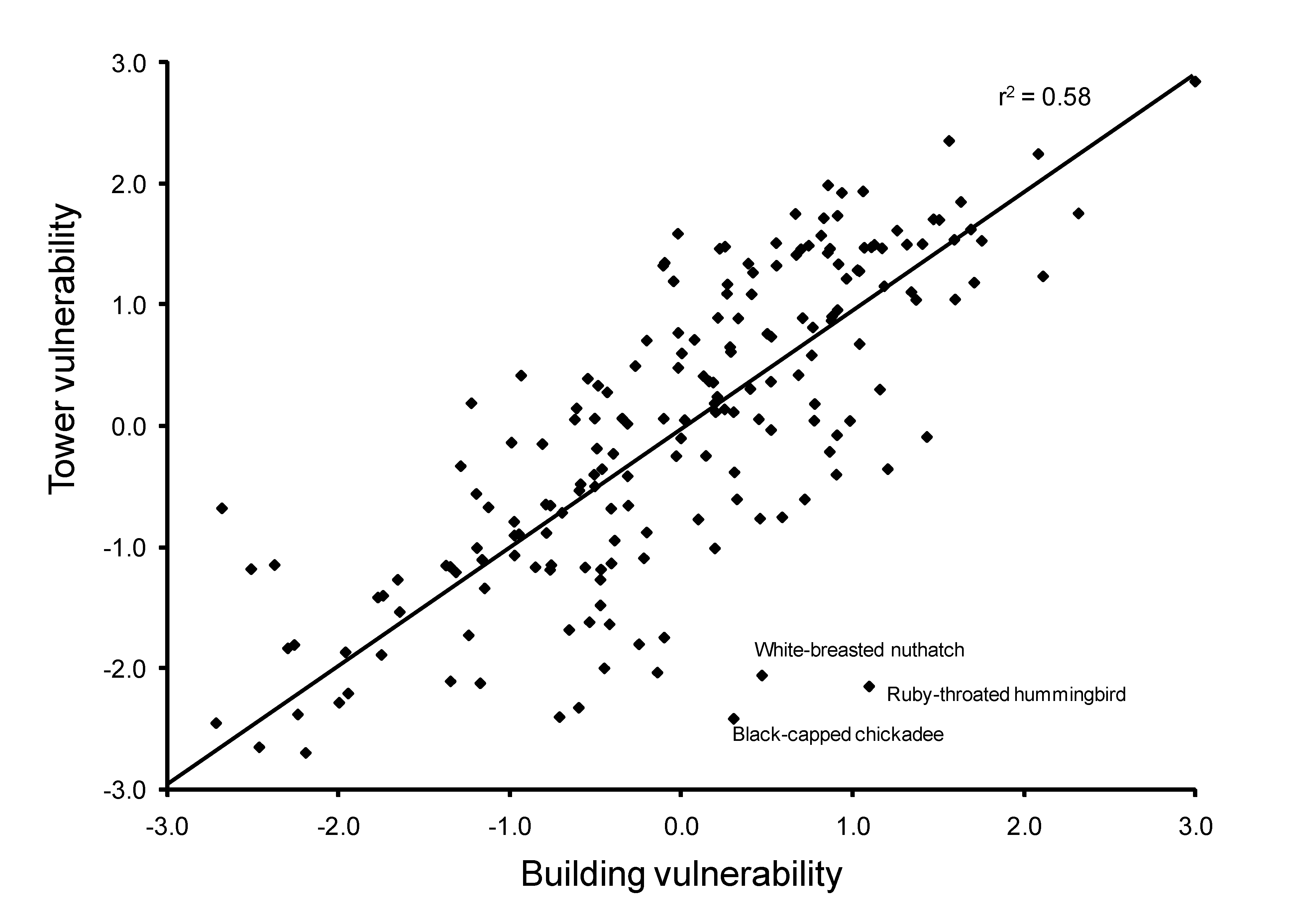

Supplement: Figure S2 — Relative vulnerability to collision with towers (i.e., residuals from Fig. 1a ) versus buildings ( Fig. 1b ). With the exception of 3 labeled outliers that had modest vulnerability to buildings but avoided towers, collision risk at towers was a good proxy for collision risk at buildings, and vice versa. (TIFF) [file pone.0024708.s002.tif]
