# Supplementary material for: Collision Mortality Has No Discernible Effect on Population Trends of North American Birds
Source: PLoS One. 2011 Sep 9;6(9):e24708. doi: 10.1371/journal.pone.0024708 (PMC3170378; doi:10.1371/journal.pone.0024708)
Supplement: Table S1 — List of North American landbird species that collide most frequently with towers (T1-T5) and buildings (B1-B5) (the “super colliders”), as well as the top five super avoiders (T188-184, B147-143). (PDF) [file pone.0024708.s003.pdf]

Table S1: List of North American landbird species that collide most frequently with towers (T1-T5) and buildings (B1-B5) (the "super colliders"), as well as the top five super avoiders (T188-184, B147-143).

Rank	Common name	Scientific name	Family	Migrant	Collisions	Pop. size	Resid	Risk	Trend
T1	Bay-breasted warbler	*Dendroica castanea*	Parulidae	LD-N	10,396	2,700,000	2.37	236	-0.1
T2	Golden-winged warbler	*Vermivora chrysoptera*	Parulidae	LD-U	542	210,000	2.29	196	**-2.3**
T3	Connecticut warbler	*Oporornis agilis*	Parulidae	LD-U	2,624	1,080,000	2.23	171	-0.8
T4	Bachman's sparrow	*Peucaea aestivalis*	Emberizidae	SD-U	74	300,000	2.01	103	**-2.8**
T5	Black-thr. blue warbler	*Dendroica caerulescens*	Parulidae	LD-U	2,061	2,000,000	1.98	96	**2.4**
B1	Swamp sparrow	*Melospiza Georgiana*	Emberizidae	SD-N	3,941	4,500,000	1.76	57	0.7
B2	Brown creeper	*Certhia americana*	Certhiidae	SD-U	1,039	2,000,000	1.68	48	**2.7**
B3	Black-thr. blue warbler	*Dendroica caerulescens*	Parulidae	LD-U	588	1,800,000	1.61	41	**2.4**
B4	Nelson's sparrow	*Ammodramus nelson*	Emberizidae	SD-N	44	100,000	1.41	26	0.2
B5	Fox sparrow	*Passerella iliaca*	Emberizidae	SD-N	1,819	4,800,000	1.40	25	1.5
T188	Horned lark	*Eremophila alpestris*	Alaudidae	SD-D	0	49,500,000	-2.84	688	**-2.9**
T187	American robin	*Turdus migratorius*	Turdidae	SD-D	6	217,000,000	-2.69	495	**0.3**
T186	Cliff swallow	*Petrochelidon pyrrhonota*	Hirundinidae	LD-D	1	32,000,000	-2.42	261	**-1.6**
T185	Black-capped chickadee	*Poecile atricapillus*	Paridae	Non-D	0	23,800,000	-2.38	240	**1.7**
T184	Common grackle	*Quiscalus quiscula*	Icteridae	SD-D	4	67,900,000	-2.35	222	**-1.9**
B147	Horned lark	*Eremophila alpestris*	Alaudidae	SD-D	0	19,800,000	-2.32	208	**-2.9**
B146	Lapland longspur	*Calcarius lapponicus*	Calcariidae	SD-B	1	35,000,000	-2.26	183	NA
B145	Cliff swallow	*Petrochelidon pyrrhonota*	Hirundinidae	LD-D	0	16,000,000	-2.22	168	**-1.6**
B144	Northern mockingbird	*Mimus polyglottos*	Mimidae	Non-D	0	14,800,000	-2.19	155	**-0.6**
B143	Tree swallow	*Tachycineta bicolor*	Hirundinidae	SD-D	0	10,000,000	-2.02	105	**-1.4**

Migrant status includes distance: long-distance (LD), short-distance (SD) or non-migratory (Non) and timing: nocturnal (N), diurnal (D), both (B) or unknown (U) [27,28]. Collisions are number of observed mortalities for each species. Population size [12] is adjusted to eastern North America. Residual is the deviation from the regression of $\log_{10}$(collisions + 1) on $\log_{10}$ population size; site overlap was also included for tower mortality. For super colliders (top 10 species) risk is 10^{Resid} and reflects the risk multiplier (e.g. bay-breasted warblers are at 236 times greater risk than the average bird); for super avoiders risk is 10^{-Resid} and reflects lower vulnerability (e.g. horned larks are 688 times less likely to collide with a tower than the average bird). Trend is the long-term (1966-2009) population trend expressed as percent change per year [14]; values in bold are statistically significant.
