# Supplementary material for: Collision Mortality Has No Discernible Effect on Population Trends of North American Birds
Source: PLoS One. 2011 Sep 9;6(9):e24708. doi: 10.1371/journal.pone.0024708 (PMC3170378; doi:10.1371/journal.pone.0024708)
Supplement: Table S2 — Taxonomic variation in collision risk for families of North American birds. (PDF) [file pone.0024708.s004.pdf]

Table S2: Taxonomic variation in collision risk for families of North American birds.

	Collision risk with towers				Collision risk with buildings			
Family	Mean	SE	n	t	Mean	SE	n	t
Hirundinidae	-1.73	0.19	6	**-8.93**	-1.67	0.20	6	**-8.25**
Strigidae	-0.86	0.09	5	**-9.23**	-0.09	0.21	5	-0.41
Picidae	-0.63	0.25	7	-2.48	-0.04	0.27	7	-0.15
Fringillidae	-0.58	0.35	8	-1.65	-0.73	0.31	5	-2.37
Icteridae	-0.53	0.31	11	-1.70	-0.57	0.19	8	-2.93
Accipitridae	-0.33	0.19	13	-1.73	-0.27	0.15	11	-1.75
Turdidae	-0.12	0.53	7	-0.23	0.26	0.26	7	1.01
Troglodytidae	0.02	0.40	6	0.05	0.37	0.23	4	1.63
Tyrannidae	0.19	0.21	10	0.90	-0.18	0.13	8	-1.34
Emberizidae	0.28	0.20	20	1.38	0.65	0.19	14	3.45
Cardinalidae	0.47	0.31	8	1.48	0.13	0.59	4	0.22
Vireonidae	1.07	0.21	8	**5.00**	-0.31	0.23	5	-1.39
Parulidae	1.26	0.10	36	**12.51**	0.58	0.10	21	**5.58**

Means and standard errors (SE) of residual collision risk (see Supplementary Table S1) averaged over all *n* species within each family for families with 4 or more species. Student's *t* tests the null hypothesis that collision risk within each family is equal to the overall average (0). Values in bold text are significant at $P < 0.05$ after applying a Bonferonni adjustment to maintain an overall error rate of 0.05.
