# Supplementary material for: Collision Mortality Has No Discernible Effect on Population Trends of North American Birds
Source: PLoS One. 2011 Sep 9;6(9):e24708. doi: 10.1371/journal.pone.0024708 (PMC3170378; doi:10.1371/journal.pone.0024708)
Supplement: Appendix S1 — Supplementary Analyses. (PDF) [file pone.0024708.s005.pdf]

Appendix S1: Supplementary Analyses.

Arnold TW, Zink RM. Collision Mortality Has No Discernible Effect on Population Trends of North American Birds.

Representativeness of collision data from towers: We did not have access to raw data from all 39 tower sites included in Shire et al.'s review, but for six tower sites comprising 196,357 total mortalities [S1-S6], counts of mortalities by species were significantly positively correlated between all pairs of sites, with an average correlation of $r = 0.53$ (range $r = 0.21$ between Eau Claire, WI and Bithlo, FL [the two most distant sites] to $r = 0.94$ between central Illinois and Nashville, TN [the two closest sites]). These six studies include most of the east-west and north-south gradient summarized in the composite data of Shire et al. [13], and we therefore conclude that relative vulnerability indices as calculated in our analyses are likely to be positively correlated with mortality data from any given tower and strongly correlated with the true composite mortality rate from all tower collisions.

Potential biases in carcass counts: Our methods do not require that all mortalities are identified, only that the proportion of mortalities identified at each collision site does not differ systematically among species (e.g. if 50% of all mortalities are found and recorded, on average, then we assume that ~50% of cerulean warblers are found, ~50% of downy woodpeckers are found, etc.). Biases could occur if some species are more likely than other species to be overlooked (i.e. carcasses are more cryptic), to be scavenged, or to be mortally wounded and able to move out of the survey area before death. We reasoned, all other things being equal, that large or colorful birds would be easier for observers to detect. We ranked each species based on size (total body length) and plumage conspicuousness (subjective scores ranging from 0 to 2, where cryptic species such as sparrows were scored as 0, shrikes and northern cardinals were scored as 2, and birds with some color or contrast were scored as 1). Relative collision vulnerability was not a function of either body size or plumage conspicuousness, as all correlations were negative ($r = -0.22$ to -0.10). Carcasses of larger and more colorful birds might be easier to find, but such biases are not the primary driving force of our vulnerability metric.

Formalized studies of scavenging rates (i.e., carcass disappearance) have found that bird carcasses persisted for an average of 6.7 to 8.6 days below communication towers [S7], 5.9 to 6.3 days beneath wind turbines [S8], and 2.1 to 4.2 days in a variety of habitats in South Dakota [S9]. Osborn et al. [S8] found no effect of body size on carcass persistence. Most studies included in our analyses involved standardized daily search protocols, minimizing the potential for scavengers to remove substantial fractions of birds. Scavenging undoubtedly led to underestimation of total mortality at collision sites, but there is no basis for supposing that differential scavenging could have affected species vulnerability estimates by even 2-fold, whereas the inferences we make about collision mortality are based on differences that exceed 10-fold in magnitude.

At Toronto, volunteers separately recorded birds found dead versus birds found injured. We used only the mortality data in our analysis, but note that the two counts were highly correlated across species ($r = 0.954$), suggesting limited potential for bias due to injured birds escaping detection. In other words, birds found wounded in large numbers were essentially identical in species composition to those found dead. It is possible that resident birds would have been more prevalent in our summary if data collection had occurred at all sites throughout the entire year, but virtually no mortality was detected during non-migratory periods for the one study that employed year-round searches [S2].

Potential biases in population trends: Correlations between relative collision vulnerability and population trends did not change if we based analyses only on population trends that were of medium to high credibility (towers: $r = -0.003$, $n = 163$; buildings: $r = 0.84$, $n = 138$) or high credibility (towers: $r = 0.030$, $n = 125$; buildings: $r =$

0.199, n = 102). Credibility measures are primarily a function of abundance and coverage, with abundant and widespread species having more reliable trend estimates than species that are rare or have restricted ranges [14].

For building data, which were more limited and not widely dispersed throughout eastern North America, we also conducted restricted analyses based on average population trends in Bird Conservation Regions (BCR) 13 (Toronto), 22 (Chicago) and 30 (New York City), correlating relative collision vulnerability at each location with population trends derived only from that BCR. Such correlations were significantly positive for Toronto (r = 0.201, P = 0.03, n = 123) and non-significantly negative for Chicago (r = -0.167, P = 0.10, n = 90) and New York City (r = -0.048, P = 0.61, n = 110), but in all 3 cases adjusted $r^2 \leq 0.03$.

Phylogenetic Analyses: For analyses involving population trends, we treated species as statistically independent data points. Species are the unit of conservation concern, and we reasoned that neither population trajectories nor collision with anthropogenic structures are traits possessing phylogenetic inertia. However, species cannot be treated as statistically independent for analyses examining ecological or behavioral correlates of collision vulnerability, because these traits (e.g. migration distance and timing of migration) are potentially confounded by phylogenetic inertia. We therefore utilized phylogenetic regression [S10] to examine collision vulnerability in relation to migration distance (long-distance, short-distance or partial/non-migrant) and migration timing (mostly nocturnal, mostly diurnal or both). The composite phylogeny we used was based on the higher-level phylogenetic hypotheses of Barker et al. [S11] and Hackett et al. [S12], combined with several family-level analyses. Branch lengths were unknown and were assigned using the default method [S10].

S1. Clark, AR, Bell CE, Morris SA (2005) Comparison of daily avian mortality characteristics at two television towers in western New York, 1970-1999. Wilson Bull 117: 35-43.

S2. Crawford, RL (1981) Bird casualties at a Leon County, Florida TV tower: a 25-year migration study. Bull Tall Timbers Research Station 22.

S3. Goodpasture KA (1984) Television tower casualties, Nashville, Tennessee 1976-1983. Migrant 55: 53-57.

S4. Kemper C (1996) A study of bird mortality at a west central Wisconsin TV tower from 1957-1995. Passenger Pigeon 58: 219-235.

S5. Seets JW, Bohlen HD (1977) Comparative mortality of birds at television towers in central Illinois. Wilson Bull 89: 422-433.

S6. Taylor WK, Anderson BH (1973) Nocturnal migrants killed at a central Florida TV tower; autumns 1969-1971. Wilson Bull 85: 42-51.

S7. Gehring J, Kerlinger P, Manville II AM (2009) Communication towers, lights, and birds: successful methods of reducing the frequency of avian collisions. Ecol Applications 19: 505-514.

S8. Osborn RG, Higgins KF, Usgaard RE, Dieter CD, Neiger RD (2000) Bird mortality associated with wind turbines at the Buffalo Ridge Wind Resource Area, Minnesota. Amer Midl Natur 143: 41-52.

S9. Kostecke RM, Linz GM, Bleier WJ (2001) Survival of avian carcasses and photographic evidence of predators and scavengers. J Field Ornithol 72: 439-447.

S10. Grafen A. (1989) The phylogenetic regression. Phil Trans R Soc Lond B 326: 119-157.

S11. Hackett SJ et al. (2008) A phylogenomic study of birds reveals their evolutionary history. Science 320:1763-1768

S12. Barker KF, Cibois A, Schikler P, Feinstein J, Cracraft J (2004) Phylogeny and diversification of the largest avian radiation. Proc Nat Acad Sci 101:11040-11045
